# Possible Future Monoclonal Antibody (mAb)-Based Therapy against Arbovirus Infections

**DOI:** 10.1155/2013/838491

**Published:** 2013-08-22

**Authors:** Giuseppe Sautto, Nicasio Mancini, Giacomo Gorini, Massimo Clementi, Roberto Burioni

**Affiliations:** Laboratorio di Microbiologia e Virologia, Università Vita-Salute San Raffaele, 20132 Milano, Italy

## Abstract

More than 150 arboviruses belonging to different families are known to infect humans, causing endemic infections as well as epidemic outbreaks. Effective vaccines to limit the occurrence of some of these infections have been licensed, while for the others several new immunogens are under development mostly for their improvements concerning safety and effectiveness profiles. On the other hand, specific and effective antiviral drugs are not yet available, posing an urgent medical need in particular for emergency cases. Neutralizing monoclonal antibodies (mAbs) have been demonstrated to be effective in the treatment of several infectious diseases as well as in preliminary *in vitro* and *in vivo* models of arbovirus-related infections. Given their specific antiviral activity as well-tolerated molecules with limited side effects, mAbs could represent a new therapeutic approach for the development of an effective treatment, as well as useful tools in the study of the host-virus interplay and in the development of more effective immunogens. However, before their use as candidate therapeutics, possible hurdles (e.g., Ab-dependent enhancement of infection, occurrence of viral escape variants) must be carefully evaluated. In this review are described the main arboviruses infecting humans and candidate mAbs to be possibly used in a future passive immunotherapy.

## 1. Introduction

Arthropod-borne virus (arbovirus) infections are increasingly becoming an emerging medical problem mostly affecting endemic areas such as developing countries or upcoming economies (like China and India). In particular, the major outbreak source of arbovirus-related diseases in endemic areas is mostly related to the presence of the viruses in an animal reservoir and a following expansion in humans. Moreover, epidemic episodes, which occur mainly during seasons with increased disease activity or outbreaks (e.g., because of climate variations), have also been described. In addition, increasing traveling to exotic and medically high-risk locations has enlarged this problem also to previously non-endemic areas, due to the global rise of travelers and movement of large populations [1].

Of the over 545 suspected arbovirus species, the most known virus-transmitting arthropods (vectors) are mosquitoes (mostly female *Aedes aegypti* and *Aedes albopictus*), ticks, midges, and sandflies. Humans are usually dead-end hosts, as they do not develop the high viremia required to infect arthropods that is sustained by vertebrate animal reservoirs [1]. 

Although several arboviruses of clinical significance in humans are known (more than 150), only a restricted group of them is globally diffused, the majority of which are zoonotic and belong to the Flaviviridae, Bunyaviridae, or Togaviridae families, with a small number belonging to Reoviridae and Orthomyxoviridae [1]. Highly effective vaccines for several of them are available, including tick-borne encephalitis (TBEV) [2], yellow fever (YFV) [3], and Japanese encephalitis (JEV) viruses [4], but for no one of them a specific antiviral drug is currently approved for clinical use. In the course of viral infections, neutralizing monoclonal antibody (mAb)-based therapy represents a promising and safe alternative strategy, in particular when a specific and efficacious treatment is not yet available [5–11]. At present, human mAb-based passive immunotherapies for arbovirosis are at very early stage of development. However, previous studies in mice have shown that passive transfer of either monoclonal or polyclonal Abs can be protective against homologous or heterologous dengue virus (DENV) challenge as well as against other *flaviviruses* and human arboviruses. Moreover, engineering rendering mAbs capable of crossing the blood-brain barrier in order to limit viral dissemination within CNS may be considered.

Finally, a possible administration of mAbs in those subjects that could be at risk of exposure to arbovirus infections, such as travelers in endemic areas, could reduce the possible incidence and consequent augmented risk of epidemic episodes. 

In this review, we describe the major clinical relevant and worldwide diffused arboviruses infecting humans and the recently major described mAbs to be possibly used in a future passive immunotherapy. 

## 2. *Flaviviruses*

The *Flavivirus* genus, including more than 70 viruses, is the only one within the Flaviviridae family which holds arboviruses that are responsible for significant morbidity and mortality worldwide [12].

About 2.5 billion people are at risk of infection in tropical and subtropical countries, mainly South-East and South Asia, Central and South America, and the Caribbean. In addition, multiple *Flavivirus* infections have been reported in the same areas, complicating early diagnosis and identification [13].

*Flavivirus* infections can cause fever, encephalitis, hemorrhagic disease, flaccid paralysis, and death in humans. However, the immunopathogenesis of these viruses is not fully understood. In the last decade, the *flaviviruses* have reemerged as aggressive human pathogens [13].

The human *flaviviruses* includes 53 recognized species. However, five of them are considered clinically important like DENV, YFV, JEV, TBEV, and West Nile virus (WNV).

The genome of all the members of the Flaviviridae family consists of a 9.5–12.5 kb positive-sense, single-stranded RNA. They are enveloped small virions (40–60 nm in diameter) with two or more species of envelope glycoproteins (e.g., M and E proteins), which are involved in the binding and fusion processes. In particular, the precursor of the mature M protein (prM) interacts with E glycoproteins, acting as a chaperone and preventing the fusion of the virus with the membrane in the cell during egress through acidic compartments of the secretory pathway. Then, cleavage of prM by the cellular protease furin during transit through the Golgi network is a required step in the viral lifecycle that defines the transition from an immature non-infectious virus particle into an infectious form. However, immature infectious virions retaining some uncleaved prM molecules could be released [14].

The M and E glycoproteins constitute an icosahedral scaffold surrounding a nucleocapsid, which consists of the viral genome complexed with a core of approximately 30 nm composed of multiple copies of a small, basic capsid (C) protein. Binding, uptake and fusion by target cells are believed to involve clathrin-mediated and low-pH-induced endocytosis [14]. 

*Flaviviruses* can utilize multiple receptors for different cell types and host species. They are thought to firstly interact with dendritic cells through DC-SIGN and L-SIGN binding of glycans on E glycoprotein dimers. In addition, highly sulfated glycosaminoglycans (e.g., heparan sulfate) have been demonstrated to play an important role in the initial attachment of several *flaviviruses* to their target cells. Other molecules identified as possible receptors are integrins, mannose-binding receptor on macrophages, laminin-binding protein, GRP78 (BiP), and CD14 [15]. 

Uncoating and replication of the viral genome, through a minus-strand RNA intermediate, occurs in the cytoplasmic replication complexes associated with perinuclear membranes, where viral proteins are produced as part of a single long polyprotein of more than 3,000 amino acids, generating three structural (C, prM, and E) and seven non-structural (NS1, NS2A, NS2B, the viral protease NS3, NS4A, NS4B, and the RNA-dependent RNA polymerase NS5) proteins by cleavage of host and viral proteases, respectively. Progeny virions are thought to assemble by budding into an intracellular membrane compartment, probably the endoplasmic reticulum, then transited through the host secretory pathway, and released at the cell surface [16]. 

Efforts to develop effective prophylactic approaches for several clinically important *flaviviruses* are underway [17]. The crucial role of the humoral immune response against *Flavivirus* infections is well established, as infection with one serotype provides life-long protective immunity to the homologous infecting serotype and cross-protection in the first few months against the other serotypes. Conversely, individuals experiencing a secondary infection with a distinct serotype are at greater risk of severe complications, as discussed later [18]. 

Only a limited number of *Flavivirus* vaccines are available today; however, no approved antiviral drugs are yet available for their clinical use. Thus, giving the lack of vaccines as well as specific antiviral drugs, broadly cross-neutralizing mAbs, could be helpful in the development of an effective therapeutic strategy against these infections as well as in the progress towards effective immunogens.

In the next paragraphs, we describe the molecular targets of *flaviviruses*, including DENV, JEV, TBEV, WNV, YFV, and St. Louis encephalitis virus (SLEV) followed by a description of the therapeutic candidate mAbs directed against them. 

### 2.1. Domains and Functions of the Surface Envelope (E) Glycoprotein of *Flaviviruses*

The major target of the host humoral immune response and of neutralizing Abs against *flaviviruses* is represented by the envelope (E) glycoprotein, which is a 56-kDa protein and the major represented antigen on the surface of virions [19]. However, Ab response towards other structural and non-structural proteins, such as prM and NS1, respectively, has also been described [20, 21]. However, prM-specific Abs display limited neutralizing activity, while anti-NS1 Abs have been detected only during the convalescent period of a primary DENV infection but were strongly identified during the acute phase of a secondary DENV infection, suggesting that both Abs could contribute to the pathogenesis of the life-threatening dengue hemorrhagic fever/dengue shock syndrome (DHF/DSS) [22].

The crystallographic structure of the E protein of TBEV has been proposed as a model for the envelope protein of the *flaviviruses* [23].

Further studies performed through X-ray crystallography have revealed that the E protein is a type II viral fusion protein with three *β*-barrel domains (D): DI, DII, and DIII (corresponding to the antigenic domains C, A, and B, resp.) connected to the viral membrane by a helical structure called the stem anchor [24]. In its native form the E protein folds as an homodimer with an antiparallel structure and an unusual herringbone pseudoicosaedral symmetry pattern with the M protein located centrally within the symmetry, consistent with a head-to-tail configuration lying parallel to the envelope lipid bilayer. The DIII of the E glycoprotein (amino acid residues 295–395) has an immunoglobulin-like fold, contains seven *β*-strands as well as type- and subcomplex-specific neutralizing B-cell epitopes and is the proposed receptor-binding domain through four peptide loops on the solvent exposed face. Directly linked to DIII, there is the stem/transmembrane region, spanning amino acid residues 401–495 and containing regions important for oligomerization with prM protein (amino acid residues 431–449 and 450–472) [25]. DII has a long, finger-like structure and contains two extended loops that project from DI and the highly conserved and hydrophobic glycine-rich fusion loop at its tip (amino acid residues 98–110), interacting with the stem region and the endosomal membrane of the cell during the fusion process [26]. In the trimeric conformation, the hydrophobic fusion peptide is exposed in DII to mediate the fusion with the membranes of the cell. DI is a ~120 amino acid central domain, formed by a discontinuous hinge region consisting in a central *β*-barrel of eight strands that connects DII and DIII and plays a crucial role in the structural rearrangement of E from a homodimer to a trimer, which occurs on exposure to low pH of endosomes that is required for the membrane fusion process. In some *flaviviruses*, an N-linked glycosylation site is present at amino acid position 154–156 of the E protein and does not appear to be necessary for E function, but it has been associated with the increased neuroinvasiveness of WNV lineage I outbreak strains from the USA and a pH-sensitive decrease in stability of the non-glycosylated strains [27].

### 2.2. *Flavivirus* Neutralizing B-Cell Epitopes

As previously described, Abs are a significant component of the host's protective response against *Flavivirus* infections. However, virus-specific Abs have been implicated in the pathogenesis of severe clinical manifestations following a secondary DENV as well as YFV and WNV infections.

Moreover, cross-reactivity between *Flavivirus* serogroups could complicate the interpretation of diagnostic assays but could be of interest in isolating cross-reactive and cross-neutralizing mAbs. However, Ab-dependent enhancement (ADE) of infection has been described for this genus as it represents the major risk of complications when reinfection occurs. Thus, great care must be taken in evaluating neutralizing activity and possible Ab-mediated infection enhancement in the characterization of future possible therapeutic mAbs against these viruses [28]. 

However, prophylactic and therapeutic use of neutralizing mAbs for *Flavivirus* infections has been shown to be effective in animal models as reported later.

Neutralizing epitopes were found to be located in each of the three domains of the E protein and have been confirmed to be surface exposed in the high-resolution X-ray crystal structure of the pre-fusion dimer of DENV2 E glycoprotein [29].

Most potent mAbs against *flaviviruses* are directed toward epitopes on DIII. For this reason, DIII-based immunogens are under evaluation as promising subunit *Flavivirus* candidate vaccines [30, 31]. However, several cross-reactive mAbs that bind to residues from the AB loop were found to be poorly neutralizing as this loop projects toward the lipid bilayer in the mature viral particle. Conversely, lateral ridge region of DIII (e.g., BC, DE, and FG loops) is targeted by strong, serotype-specific neutralizing mAbs [32–35]. Cross-reactive mAbs specific for DII have also been described but with less neutralizing and variable profiles. However, few of them, whose epitope encompasses the fusion loop, are cross-reactive and neutralizing [36]. 

### 2.3. Ab-Dependent Enhancement (ADE) of Infection

**Figure 1:**
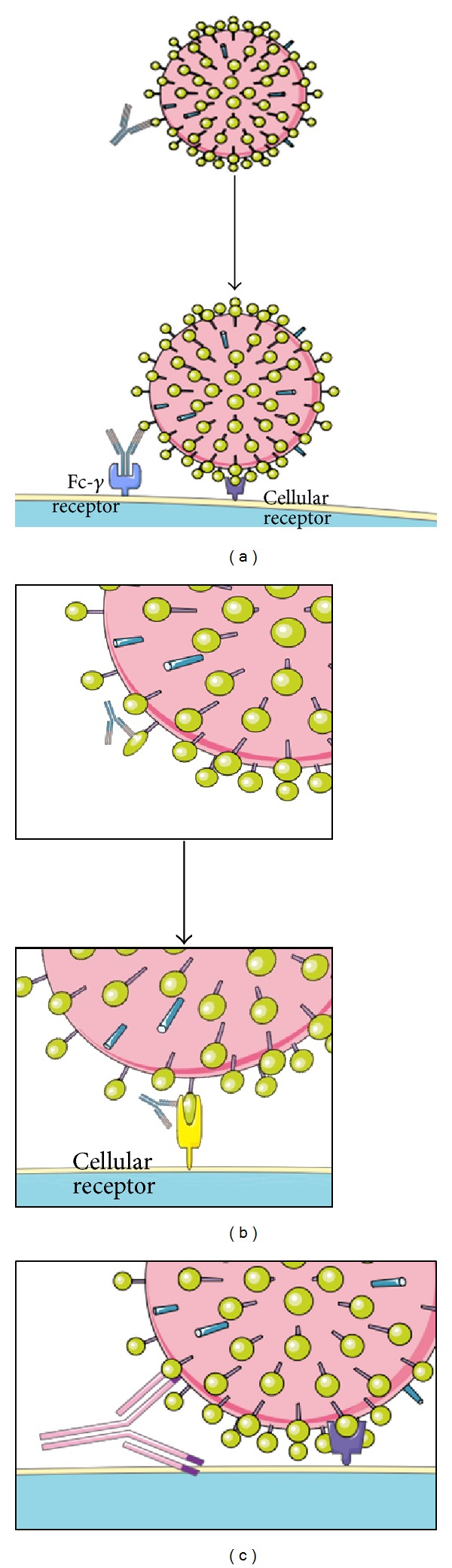
Mechanisms of antibody-dependent enhancement (ADE) of infection. (a) After binding to the viral epitope, the Ab is recognized by a cellular Fc-*γ* receptor, bringing the viral particle in proximity of the target cell; (b) the binding of the Ab induces conformational changes within the structure of the viral target protein. These changes improve the affinity for the cellular receptor; (c) molecular mimicry by a viral motif of cellular membrane components leads an autoreactive Ab to bind both the viral and the cellular target, bringing the virus in proximity of the target cell.

*In vitro* and *in vivo* studies have demonstrated that increased disease severity, causing DHF/DSS, upon reinfection with a different DENV serotype, is mostly due to the phenomenon of ADE of infection, determined by cross-reacting but poorly or non-neutralizing Abs, generated during the primary infection, that facilitate virus entry through Fc-*γ* or complement receptors present on cells, such as monocytes/macrophages (Figure 1(a)). Additionally, in 6 to 9 month-old children of DENV immune mothers, severe disease is associated with primary infection, possibly because of declining levels of neutralizing maternal Abs [64].

These cross-reactive Abs are mainly directed against the E and prM glycoproteins. In particular, experimental lines of evidence have suggested that cross-reactive Abs against DII of E protein can, under certain conditions, enhance infectivity of WNV *in vitro *[65]. However, the phenomenon of ADE could reflect the presence of non-neutralizing concentrations of virus-reactive Abs and has been observed for several *flaviviruses in vitro *and *in vivo*, such as WNV, TBEV, JEV, and YFV as well as other Flaviviridae members [28, 66–68]. In particular, it has been described that ADE of infection occurs at virus-bounded Ab concentrations in the upper limit by the stoichiometric threshold for neutralization, and at lower concentrations by the minimal number of Abs that allow attachment of the virion to cells. Furthermore, Abs that recognize infrequently displayed epitopes that do not support neutralization may enhance infection even at saturation [69].

Thus, the ADE of infection mechanism may pose a threat for the development of a safe and efficacious vaccine as well as a possible immunotherapy against Flaviviridae. 

Indeed, cross-neutralizing mAbs, as candidate therapeutics for these infections, may be considered after having well ascertained their broadly cross-neutralizing activity and the absence of possible mAb-mediated ADE of infection mechanisms. Alternatively, the risk of ADE of infection could be overtaken by the removal of the Ab heavy chain (i.e., the CH2 and CH3 portions or direct expression of the mAb as a Fab fragment) and/or deletion of the N-linked sugars on IgG molecules that are both required for interactions with Fc-*γ* receptors (Fc-*γ*R), or eventually, by the blocking of Fc-*γ*R engagement with anti-Fc-*γ*R Abs [70]. Similarly, the identification and detailed examination of cross-neutralizing epitopes that do not promote ADE of infection may define novel targets for vaccine development.

However, in some instances, ADE of infection may occur when Ab molecules cross-react with both viral and cellular antigens (Figure 1(c)). In this regard, Fc-*γ* receptor-independent mechanism of infection, in particular for DENV prM-specific Abs able to bind simultaneously to the virus and target cells, has been described. Indeed, other members of the Flaviviridae belonging to another genus, like hepatitis C virus (HCV), have been shown to elicit polyreactive Abs that are probably responsible for an ADE of infection mechanism as well as of secondary clinical manifestations. In connection with this, several studies, employing both polyclonal and monoclonal Abs, suggested the presence of common epitopes containing particular short motifs on NS1 and the three domains of the E protein as well as human proteins that may play a role in ADE of infection mechanisms and thus in DHF/DSS pathogenesis [70]. Interestingly, these common motifs have been found to be additionally represented in DENV2 strains which have greater human pathogenic capacities [22].

Moreover, another molecular mechanism of ADE of infection could consist in the facilitation of conformational changes of the targeted surface protein that are required for virus entry, as described and hypothesized for other viruses (e.g., HIV, HCV, and HSV) (Figure 1(b)) [71, 72]. 

Finally, Fc-*γ*R engagement could result also in “intrinsic” ADE of infection mechanisms involving signaling events, such as inhibition of antiviral response and increased viral replication. Disrupted signaling events include the RIG-I/MDA5 cascade and type I interferon (IFN) response as well as induction and suppression of anti-inflammatory (IL-10) and proinflammatory (IL-12 and TNF-*α*) cytokines, respectively [73].

### 2.4. Dengue Virus (DENV)

Dengue virus (DENV) is responsible for 50–100 million symptomatic infections each year, resulting in 500,000 hospitalizations and over 20,000 deaths which occurs mostly in tropical and subtropical regions of the world. Although malaria remains the most important cause for systemic febrile disease in travelers, chikungunya virus (CHIKV) and DENV are increasingly diagnosed, with dengue currently being the second most important cause for febrile disease in travelers.

Decreases in mosquito control efforts during the end of the 90s, coupled with societal factors (e.g., globalization, migrations, and dense urbanization) have contributed to the reemergence of *flaviviruses* such as DENV in South and Central America. Development of effective DENV vaccines that exhibit cross-protection, thought to be important for preventing subsequent dengue-associated immunopathogenesis, is proving to be particularly challenging [1]. 

A vaccine for this infection is not yet available despite considerable public and private efforts. This difficulty is mainly due to the perceived need to simultaneously protect the four known serotypes of DENV (DENV1–4), which share about 70% of sequence homology, while genotypes can vary up to 3%. Moreover, as anticipated and discussed previously, non-protective Abs may contribute to more severe clinical outcomes in vaccinated people [74].

#### 2.4.1. Murine versus Human Anti-DENV Humoral Immune Response

It is well ascertained that anti-E DIII-directed Abs are virtually absent from the naïve human repertoire as they are directed away from this domain, probably against weakly neutralizing and immunodominant regions. Moreover, it has been demonstrated that over than 90% of the human Ab response of primary DENV infected patients is able to bind only native DENV virus particles instead of a recombinant form of the E protein, posing difficulties in the cloning strategies of neutralizing mAbs [39]. Thus, it can be concluded that *in vivo *only a small fraction of DENV-specific Abs are responsible for neutralization.

Conversely, murine mAbs that recognize all the three domains of E have been identified. Epitope-mapping studies using murine serotype-specific mAbs have demonstrated that loops located in the lateral ridge region of DIII constitute the strongest neutralizing B-cell target. In this region, sequence diversity between serotype is high while cross-reactive murine mAbs that recognize another loop showed generally a weaker neutralization profile. In particular, type-specific mAbs with neutralizing activity against DENV2 localized to the BC, DE, and FG loops on the lateral ridge of DIII, whereas subcomplex-specific mAbs recognized an adjacent epitope centered on the connecting A strand of DIII at residues K305, K307, and K310 [32–35].

Cross-reactive mAbs directed against prM and NS1 have also been described, but, as anticipated, they feature weakly neutralizing or absent as well as ADE of infection activity. In the following paragraphs, we report the better characterized neutralizing mouse and human mAbs, describing their molecular features further summarized in Table 1.

#### 2.4.2. Anti-DENV Murine mAbs

Murine mAbs directed against all the three domains of the E protein and endowed of different neutralizing and binding characteristics have been described. However, only DIII and DII are classically recognized by neutralizing Abs. In this regard, Sukupolvi-Petty et al. isolated twenty-four anti-DENV2 mouse mAbs with moderate or strong neutralizing activity against the homologous virus in cell culture assays. Binding sites were mapped for the majority of these in distinct epitopes in regions located in DI (lateral ridge), DII (dimer interface, lateral ridge, and fusion loop), and DIII (lateral ridge, CC′ loop, and A strand). Moreover, 16 of the neutralizing mAbs were tested in mice, with most of them being protective when given as prophylaxis. Seven of these had post-exposure therapeutic activity when administered as a single dose by intraperitoneal route even 3 days after intracranial infection. For the mAbs with the greatest therapeutic potential, protection was confirmed with an Ab-enhanced vascular leakage mouse model of DENV2 infection [35].

Recently, Deng et al. described a cross-reactive murine mAb, named 2A10G6, that is able to recognize DENV1–4, YFV, WNV, JEV and TBEV, to potently cross-neutralize DENV1–4, YFV and to a lesser extent WNV. This mAb recognizes a highly conserved motif (amino acid residues 98–101) located within the fusion loop of the *Flavivirus* DII of the E glycoprotein. Moreover, this mAb exerts its neutralizing activity in a post-attachment step during the virus entry process, as demonstrated by kinetic neutralization tests performed *in vitro*. Additionally, protection experiments performed in mouse models showed that treatment with 100 *μ*g/mL of 2A10G6 conferred full protection against lethal DENV2 challenge, and 20 *μ*g/mL and 4 *μ*g/mL of 2A10G6 protected 89% and 40% of infected mice from lethal challenge, respectively. For infection with DENV1, 3, and 4, use of 100 *μ*g/mL of 2A10G6 conferred partial protection, and 53%, 77%, and 73% of the infected mice survived after challenge, respectively. Finally, the protection profile of 2A10G6 against WNV showed that prophylactic administration with a single dose of 200 mg of 2A10G6 conferred 80% protection in mice. Most importantly, 3 of 8 (37.5%) mice survived when 2A10G6 was administered one day after WNV challenge. Similarly, another mouse mAb, 4G2, is able to recognize the fusion loop at the extremity of DII from all four DENV serotypes and to prevent syncytia formation [36].

Rajamanonmani et al. described a mouse mAb, 9F12, raised against the DIII of DENV2 E protein, that is able to recognize DENV1–4 serotypes as well as WNV and to neutralize five DENV strains representative of all DENV serotypes [41]. Similarly, Cockburn et al. reported a comparative, high-resolution crystallographic analysis of an A-strand DIII murine mAb, 4E11, in complex with its target domain of the E protein from the four DENV serotypes. MAb 4E11 is capable of recognizing and neutralizing all four serotypes with IC50 values varying between 1 and 100 nM. The structures reported also highlight the mechanism by which anti-A-strand mAbs disrupt the architecture of the mature virion, inducing dimer dissociation, premature fusion-loop exposure, and concomitant particle inactivation [40]. Also, Midgley et al. functionally characterized another murine mAb, 2H12, raised in mice against DIII of E protein. Similarly to the previously described mAbs; 2H12 is able to bind all the four DENV serotypes in a epitope encompassing the conserved ^314^ETQH^317^ motif. However, the neutralizing potential of 2H12 is lower than a number of other anti-DIII mAbs, with IC50 values ranging from 0.56 to 145 nM for DENV1, 2, and 4. On the other hand, it showed no ADE of infection activity [38].

Austin et al. isolated a murine mAb, named E111, which recognize a novel CC′-loop epitope on DIII of the E protein from two different DENV1 genotypes. Docking of the mAb structure onto the available cryoelectron microscopy models of DENV virions revealed that the E111 epitope was inaccessible, suggesting that this mAb recognizes an uncharacterized virus conformation. While the affinity of binding between E111 and DIII varied by genotype, a limited correlation with neutralizing activity was observed. These data support the conclusion that potent neutralization depends on genotype-dependent exposure of the CC′ loop epitope [49]. In fact, as previously described elsewhere, binding of some E reactive Abs depends on the dynamic movement of protein molecules “breathing” in the virion particle leading to transient exposure of hidden epitopes. For instance, optimal binding of mouse mAb 1A1D-2 to EDIII requires incubation at 37°C [75]. The structure of the 1A1D-2 bound to EDIII indicates that the mAb binds to sites that are transiently exposed during viral “breathing” at 37°C and block infection during attachment of the virion to the cell. In particular, this mAb neutralizes DENV1, 2, and 3 serotypes and residues K307 and K310 are the most critical residues for binding of 1A1D-2 mAb [46, 75].

Finally, Shrestha et al. immunized mice with a genotype 2 strain of DENV1 virus and generated 79 new mAbs 16 of which strongly inhibited infection by the homologous virus and localized to DIII of E protein. Surprisingly, only two mAbs, E105 and E106, retained strong binding and neutralizing activity against all five DENV1 genotypes as well as being protective in immunocompromised infected mice. Moreover, E105 and E106 exhibited therapeutic activity even when administered as a single dose four days after inoculation with a heterologous genotype 4 strain of DENV1 in the same mouse model [32].

#### 2.4.3. Other Non-Human mAbs

Other non-human and non-murine as well as humanized mAbs directed against the E glycoprotein have been described. In this regard, Goncalvez et al. isolated a large panel of anti-E Fab fragments from chimpanzees infected with all four DENV serotypes. However, only a limited number of them displayed a cross-neutralizing activity against DENV1 and 2 and to a lesser extent against DENV3 and 4. In particular, the authors calculated that, among them, the 1A5 mAb that was further humanized, neutralized DENV1–4 at a PRNT50 titer of 0.48, 0.95, 3.2, and 4.3 *μ*g/mL, respectively. Interestingly, the humanized 1A5 was also tested for binding and neutralization against the WNV/DENV4 chimera, JEV strain SA14-14-2, and Langat virus (LGTV) strain TP 21, giving a PRNT50 titer of 3.8, 21, and 28 *μ*g/mL, respectively. Moreover, the authors calculated that when administered in a dose of 2 mg per kg of body weight 1A5 would give a serum titer of approximately 40 and 20 50% reduction in plaque reduction neutralization test (PRNT50) against DENV1 and DENV2, respectively [76]. Furthermore, epitope mapping of 1A5 mAb localized to G106 within the *Flavivirus*-conserved fusion loop in DII of DENV2 E protein [51].

#### 2.4.4. Anti-DENV Human mAbs

As anticipated, attempts to isolate neutralizing human mAbs have been more challenging due to the restricted elicited Ab-repertoire which recognizes DIII of E glycoprotein in naïve as well as in infected patients [77, 78]. However, several groups reported the isolation of neutralizing mAbs from infected subjects. In this regard, Setthapramote et al. isolated a total of 136 human hybridoma clones producing specific mAbs against DENV, obtained using PBMCs from nine blood samples from four acute-phase patients secondarily infected with DENV2 and five convalescent-phase patients. Interestingly, the authors found that most of the acute-phase mAb clones were cross-reactive with all four DENV serotypes, with most of them recognizing the E protein and endowing of neutralizing activity against all DENV serotypes, compared to those derived from convalescent-phase patients. In particular, from the acute-phase PBMCs, 81.8% were anti-E, 6.6% were anti-prM, and 3.3% were anti-NS1, while 13.3% anti-E, 13.3% anti-prM, and 53.3% anti-NS1 mAbs clones were obtained from convalescent-phase PBMCs [79]. Previous studies confirmed the data obtained by Setthapramote et al. on convalescent-phase patients, but this group firstly reported the efficient preparation of human mAbs with strong neutralizing activity titers against all four DENV serotypes using PBMCs from acute-phase patients secondarily infected with DENV [80, 81].

The group of de Alwis, through immunoglobulin depletion studies, reported that a substantial fraction of DENV-reactive Abs in human immune sera, including type-specific neutralizing Abs, bound to the intact virion but not to recombinant E protein. The authors confirmed these observations also isolating human neutralizing mAbs and proposed that humans produce Abs that neutralize DENV infection by binding a complex, quaternary structure epitope that is expressed only when E proteins are assembled on a virus particle [48]. Similar findings have been described for several other viruses, including WNV and HIV [82, 83]. Mapping studies indicate that this epitope has a footprint that spans adjacent E protein dimers and includes residues at the hinge between domains I and II of E protein [48]. 

Recently, Costin et al. isolated three broadly neutralizing anti-DENV human mAbs named 4.8A, D11C, and 1.6D. These mAbs were isolated from three different convalescent patients with distinct histories of DENV infection. All three mAbs recognized the E glycoprotein with high affinity, neutralized all four serotypes of DENV but mediated ADE of infection in Fc receptor-bearing cells at subneutralizing concentrations. Mapping studies revealed that all three mAbs bind discontinuous epitopes within the highly conserved fusion-loop region of DII (contacting residues W101, L107, and/or G109) [37].

### 2.5. Yellow Fever Virus (YFV)

The WHO estimates that there are 200,000 cases of yellow fever virus (YFV) infections and 30,000 related deaths every year especially in Sub-Saharan Africa. In fact, different to the majority of human arboviruses and similarly to DENV and CHIKV, YFV has expanded its host range to include humans as an amplifying host [1]. Symptoms occurring from 3 to 6 days after infection with YFV consist mostly in fever, chills, anorexia, lumbosacral pain, nausea, and vomiting. This syndrome lasts about 3 days in most cases and is sometimes followed by a one-day lasting period of remission. Fever will reoccur together with dark hematemesis, melena, petechiae, and other hemorrhagic symptoms. Convalescence is characterized by deep asthenia lasting up to two weeks. Rarely, prognosis can be fatal. An effective live-attenuated vaccine (17D) derived from the Asibi strain by serial passages in chicken embryos is available [84]. 

#### 2.5.1. mAbs against YFV

In 1983, Schlesinger et al. produced a battery of mAbs after immunization of BALB/c mice by injection of 17D YFV vaccine strain [84]. Among them, the specificity towards the E and NS1 proteins was described, even though anti-NS1 Abs were not able to show neutralizing activity. The 13 IgG and 1 IgM anti-E protein mAbs were classified in five groups according to their specificity. Group A, consisting of the only IgM produced in this experiment, could neutralize 17D YFV virus only; group B 17D and Asibi strains only; group C Asibi strain only; group D could only neutralize Asibi but not 17D strain, showing also reactivity against DENV2, Zika, or Banzi viruses. All of the four mAbs belonging to this group were able to cross-react with Zika and/or Banzi viruses, and two (4E11 and 5H3) neutralized DENV2 virus, with 4E11 neutralizing also Banzi virus. The 3E9 mAb, the only component of the group E, could not neutralize 17D nor Asibi strains.

All of the IgG mAbs resulted able to protect both prophylactically and therapeutically BALB/c or CD-1 mice from lethal intracerebral challenge with 17D-204 strain [85]. The chimeric form of the group B mAb 2C9 IgG (2C9-cIgG) was able to provide AG129 mice a 72% survival when administered 24 hours before infection with 17D-204 strain. Its murine form could provide the survival of the 95% of the mice. Appreciable results were also obtained when both murine 2C9 and 2C9-cIgG were administered 24 hours post-infection, with the survival of 70% and 20% of mice, respectively. Viral presence was not detected in surviving mice [60]. 

In 2005, Daffis and colleagues constructed two Ab-phage libraries by cloning the repertoire of YFV-infected patients. Panning was then performed with YFV-17D virions. The scFv-5A, 7A and R3(27) showed a neutralizing activity spanning from 50% to 100% in PRNT assays against both YFV 17D-204-WHO and Asibi strains. In further tests, reactivity was observed against wild-type YFV strains of West Africa genotype I and II (Nigeria 1987 and Asibi strains, resp.), and East/Central Africa (CAR 1986, Ethiopia 1961 strains). A concentration from 0.1 to 0.3 *μ*g/mL could yield a 90% plaque reduction. Reactivity was also observed against the strain Senegal 1990 in a lesser extent. Production of escape variants could demonstrate that scFv-5A, 7A and R3(27) epitopes are built up by residues extensively separated in the monomers of E glycoprotein, that however result in closed proximity when the homodimeric form of E is constituted [61]. 

### 2.6. West Nile Virus (WNV)

The West Nile virus (WNV) is an epidemic neurotropic virus estimated to be responsible of about 36,000 cases and 1,500 deaths registered in the United States between 1992 and 2012 [86]. WNV antigenicity allows its classification into the Japanese encephalitis virus (JEV) serocomplex. Genomic analysis has revealed two main genetic lineages of WNV: lineage I viruses, circulating in the USA, Europe, the Middle East, Africa, India, and Australia, and lineage II viruses, isolated from Sub-Saharan Africa and Madagascar [1].

WNV was firstly identified in 1937 and is endemic in many countries of Africa, Middle East and West Asia. However, after 1990 frequent outbreaks of WNV infections were reported in Romania, Israel and later in North America and across the USA in 1999 and recently, in 2012 [87].

Most of WNV infected individuals do not develop symptoms while about 20% develop a self-limiting illness called West Nile fever. Acute symptoms include fever, tiredness and swollen lymph glands but in a minority of cases also encephalitis with long-term deficits in cognitive function and motor skills have been reported. However, in less than 1% of infected patients, WNV having crossed the blood-brain barrier, is responsible of neuroinvasive and potentially lethal form of the disease. In these cases, degeneration and apoptosis upon infection of neurons and the consequent inflammatory response can occur. Related symptoms include high fever, coma, muscle weakness, and paralysis. Immunodepression and advanced age have been correlated with an augment risk to develop a severe disease [87]. There are no specific treatment options or licensed vaccines for humans [88].

WNV is transmitted by infected mosquitos and initial replication is thought to occur primarily in dendritic cells in the skin, which migrate to secondary lymphoid tissues where the replicating virus enters the circulation. To date, only efficacious WNV veterinarian vaccines have been licensed, while there are no licensed vaccines for protection against WNV in humans [88]. 

#### 2.6.1. mAbs against WNV

Broad-spectrum antivirals, such as type I IFN-*α*, ribavirin, mycophenolic acid, in WNV infection showed ineffective results *in vivo* despite *in vitro* they showed some activity [89]. 

However, experiments in murine models, extrapolation of clinical data as well as passive administration of pooled immune-*γ*-globulins (OmriGam), containing a significant titer of neutralizing Abs, before and after infection, showed an important role of the humoral immune response in controlling viremia and prevent viral dissemination. Indeed, the development of a neutralizing mAb-based therapy seems to be encouraging for a possible treatment of infected patients [90–92].

Similarly to what has been observed for DENV, only anti-E Abs have been identified as neutralizing and protective. Also anti-NS1 Abs have shown a protective role, however, their mechanism of protection has not yet been elucidated, as the NS1 protein is secreted from infected cells and not present on the virions [93–95]. 

In this regard, similarly to what has been described for DENV, studies performed using a naïve human scFv library for panning with purified WNV E protein, support the hypothesis that no Abs against the neutralizing DIII can be isolated. However, DIII specific Abs were isolated in a subsequent study using immunoglobulin libraries obtained from three WNV infected patients for biopanning on purified inactivated virus, virus-like particles consisting of prM and E proteins or recombinant E glycoprotein. Although the proportion of DIII-specific mAbs was low (8%) compared with anti-DII mAbs (47%). Two out of the four anti-DIII mAbs were potently neutralizing and protective *in vivo*, whereas only three out of the 24 anti-DII were weakly neutralizing *in vitro* and non-protective *in vivo*. In particular, residues that are critical for neutralization lies on the regions spanning amino acids 305–312, 330–333, and 365 that are located on adjacent exposed loops of DIII. However, sequence alignment of E protein of different *flaviviruses*, such as DENV and JEV, revealed a considerable variation compared to the whole E protein. This observation further suggests that differently from broadly cross-reactive anti-DII Abs, anti-DIII neutralizing Abs are virus-type specific. However, of these mAbs, two (CR4374 and CR4353) protected mice from lethal WNV challenge at 50% protective doses of 12.9 and 357 *μ*g/kg of body weight, respectively [50].

Sánchez et al., using three different immunization strategies (i.e., inactivated virus, naked DNA, and recombinant protein), isolated nine murine mAbs, most of which bound to conformation-dependent epitopes in DIII of the E protein. In particular, neutralizing mAbs, named 8B10, 11C2, 10C5, and 17C8, were obtained from mice immunized with inactivated virus alone or in combination with a DNA plasmid and bound to the same region of DIII with high affinity. In contrast, mAbs obtained by immunization with a soluble version of the E glycoprotein did not exhibit neutralizing activity. These non-neutralizing mAbs were cross-reactive with several other *flaviviruses*, including SLEV, JEV, YFV, and Powassan virus, confirming the conserved nature of *Flavivirus* non-neutralizing epitopes [96].

Gould et al., isolated 11 unique human single-chain variable region Ab fragments (scFvs) that bind the E protein of WNV. Among them, a human mAb, named mAb11, expressed as a scFv-Fc fusion protein was further characterized. It recognizes the fusion loop, at the distal end of DII of the WNV E protein and cross-reacts with all four DENV serotypes, and provides protection against DENV2 and 4 as well as WNV [42]. Moreover, therapeutic studies of this mAb in WNV-infection model mice provided substantial protection when administered after 5 days post-infection. Interestingly, a neutralization escape variant of this mAb failed to cause lethal encephalitis (at higher infectious doses) or induce the inflammatory responses associated with blood-brain barrier permeability in mice, compared to the parental WNV, suggesting an important role for the fusion loop in viral pathogenesis [43].

Oliphant et al., isolated an anti-DIII E protein mAb, named E16, from hybridomas obtained after immunization of mice with recombinant WNV E protein, which neutralized all WNV strains with PRNT50 values of 4 to 18 ng and PRNT90 values of 53 to 297 ng. One hundred micrograms of mAb protected greater than 90% of mice from lethal infection and even a single 4 *μ*g treatment of E16 on day 2 after infection prevented mortality. Moreover, humanization of this mAb confirmed as therapeutically effective in mice [44]. Subsequent studies revealed that mAb E16 neutralization is mediated by engagement of four discontinuous segments of DIII including the amino-terminal region (amino acid residues 302–309) and the three connecting loops BC (amino acid residues 330–333), DE (amino acid residues 365–368), and FG (amino acid residues 389–391). Moreover, no ADE of infection was detected when E16 mAb was used at saturating concentrations [45, 47]. 

Furthermore, results of a Phase I safety study of the humanized E16 mAb (designated MGAWN1) have been reported and suggested that doses of up to 30 mg/kg were well tolerated with few mild adverse events and would provide an excess of virus neutralizing activity for 3-4 weeks after treatment. However, a case of anti-MGAWN1 Ab elicitation occurred with the consequent increased rate of clearance and indeed impacting efficacy. A Phase II safety and efficacy study of MGAWN1 is ongoing [97]. Furthermore, the possibility of preventing or treating WNV-induced memory deficits was recently investigated. In this study, hamsters were treated intraperitoneally with 32 mg/kg of MGAWN1 mAb at 4.5 days after subcutaneously challenging with WNV. Interestingly, MGAWN1 prevented mortality, weight loss and improved food consumption of WNV-infected hamsters compared to controls [98].

Recently, Lelli et al. isolated six anti-E mAbs from inactivated-WNV immunized mice. In particular, three of them (3B2, 3D6 and 4D3) neutralized lineage I and II WNV, with the first two recognizing the same epitopes located on the distal lateral surface of DIII (critical amino acid residue K307). Conversely, 4D3 mAb recognized a novel neutralizing epitope on DII (critical residues S276 and T278). Indeed, further protective and therapeutic studies are needed to ascertain their neutralizing activity *in vivo *[99].

Finally, to conclude, like DENV infection, Abs directed against the M protein are not protective and neutralizing. Moreover, in humans, a skewed humoral immune response against DII has been frequently observed and confirmed also with the hybridoma technology [50]. The isolation and elicitation of neutralizing Abs directed against the fusion loop and DIII of the E protein represent thus the most challenging and promising goal for the development of new effective therapeutic inhibitors and immunogens, respectively. 

### 2.7. Tick-Borne Encephalitis Virus (TBEV)

Tick-borne encephalitis virus (TBEV) is one of the most dangerous agents causing human neuroinfections, occurring mostly in Europe and Asia, and with a potential fatal prognosis [100]. TBEV is believed to cause 3,500–10,000 human cases of encephalitis in Europe per year, with a high morbidity in Russia, Czech Republic, Austria, and Germany. In particular, between 1990 and 2007, an average of 8,755 cases of TBE was reported per year in Europe and Russia. Despite the fact that Russia is the country with most infections registered annually, Czech Republic incidence is among the highest in Europe, with 400–1,000 clinical cases reported every year [101].

Three subtypes of TBEV are classified, namely, European, Siberian, and Far-Eastern, sharing most of the genetic and antigenic features [102]. In fact, a high degree of antigenic homogeneity between different strains of TBEV has been described [103].

Clinical manifestations vary among the subtypes, but usually they start with a short febrile period of 7–14 days after the tick bite. Fatigue, headache, and pain in the neck, shoulders, and lower back, together with high fever and vomiting may be present [100]. These manifestations are often followed by an asymptomatic phase lasting from 2 to 10 days after remission from the fever, with possible progression to neurological disease. Neurological symptoms include meningitis, encephalitis, myelitis, and radiculitis. Mortality occurs in 1-2% of the European subtype-infected patients, but fatal prognosis can occur in up to 20–40% of the Far-Eastern subtype-infected patients [104]. Mortality rates of the Siberian subtype are similar to those observed for the European [105].

Effective and safe vaccines against TBEV produced from inactivated virus have been developed and licensed for their use in humans. However, an emergency therapy in the absence of a mass immunization is needed as no effective treatments are yet available [106].

#### 2.7.1. mAbs against TBEV

Levanov et al. described the chimerization of two murine mAbs (13D6 and 10C2) directed against the DIII and DII, respectively, of the TBEV E glycoprotein. The chimeric mAbs present binding characteristics similar to the parental mAbs. Moreover, as the parental mAbs, only the chimeric mAb 13D6 was able to neutralize TBEV infectivity *in vitro*. In particular, neutralization studies with the murine, chimeric, and scFv forms of mAb 13D6 were performed. In particular, murine 13D6 showed an IC50 titer of 11.5 *μ*g/mL in Focus Reduction Neutralization Tests (FRNT) against TBEV strain 205 and of 2.9 *μ*g/mL in PRNT against TBEV strain Softjin. Chimeric 13D6 showed an IC50 titer of 4.5 *μ*g/mL in FRNT against TBEV strain 205 and of 1.9 *μ*g/mL in PRNT against TBEV strain Softjin. ScFv 13D6 showed an IC50 titer of 16.7 *μ*g/mL in FRNT against TBEV strain 205 and of 11.2 *μ*g/mL in PRNT against TBEV strain Softjin [62].

### 2.8. Japanese Encephalitis Virus (JEV)

Japanese encephalitis virus (JEV) is a mosquito-borne *flavivirus* that over the past few decades has caused several outbreaks throughout China, Southeast Asia, Australia, and Papua New Guinea with a prevalence recently estimated to be of about 70,000 cases/year [107–109]. A 40% mortality was recorded in some of the JEV-affected areas. Moreover, many survivors face some neurological problems and complications [108]. Since 1995, the disease has also emerged in Non-Asian regions such as Northern Australia [110, 111]. The situation in Southeast Asia, however, is further complicated by the overlapping epidemics of JEV and DENV as well as sporadic cases of WNV infections detected in some of the affected areas particularly in India [112].

Effective vaccines, both live-attenuated and inactivated strains of JEV, have been developed and licensed in major countries. However, as for the other arthropod-borne *Flavivirus* members, no specific antiviral drugs are currently available [4]. 

#### 2.8.1. mAbs against JEV

As previously described for the other *Flavivirus* members, many groups isolated and characterized anti-JEV mAbs showing different neutralizing and protective properties. In this regard, Gupta et al. used combinations of anti-E JEV mAbs (Hs1-4) in mice protection experiments. In particular, they found that the singularly mAb protection ranged from 45 to 65% when 100 *μ*g of mAb were administered, while equimolar combinations of two or three mAbs gave 85–90% or 100% protection, respectively [113]. In similar experiments, Lee et al. demonstrated that a *Flavivirus* anti-NS1 mAb, named 16NS1, cross-reacted with JEV as well as WNV and exhibited protective activity against WNV as well as a lethal JEV infection. However, no neutralizing activity was observed using this mAb against both WNV and JEV in *in vitro* experiments, suggesting the participation of other Ab-mediated mechanisms *in vivo*. In particular, 95% of mice were protected when 500 *μ*g of mAb were administered intraperitoneally and concomitantly to intramuscular injection of JEV. 

Overlapping peptide mapping analysis combined with site-specific mutations identified the ^116^KAWGKSILFA^125^ region and critical amino acid residues, W118 and I122, as 16NS1 mAb epitope, highly conserved in WNV and JEV strains [114]. 

Arakawa et al. isolated from a combinatorial human Fab library constructed from peripheral blood lymphocytes obtained from JEV hyperimmune volunteers. Among 188 randomly selected clones, FabTJE12B02 showed the best 50% focus reduction endpoint at the concentration of 50.2 *μ*g/mL against the JEV strain Nakayama [55]. 

Goncalvez et al. isolated three mAbs, named Fabs A3, B2, and E3, by repertoire cloning from chimpanzees initially immunized with inactivated JE-VAX and then boosted with attenuated JEV SA14-14-2. In particular, these mAbs reacted with epitopes in three different E domains: in DI (amino acid residue K179), in DII (I126), and in DIII (G132) for Fabs A3, B2, and E3, respectively. Moreover, these Fabs as well as the derived humanized counterpart mAbs exhibited high neutralizing activities against a broad spectrum of JEV genotype strains. Moreover, these mAbs exhibited a 50% protective dose of 0.84 *μ*g (B2), 5.8 *μ*g (A3), and 24.7 *μ*g (E3) in mouse models. Finally, administration of 200 *μ*g/mouse of mAb B2 one day after otherwise lethal JEV infection protected 50% of mice and significantly prolonged the average survival time compared to that of mice in the unprotected group [54].

### 2.9. St. Louis Encephalitis Virus (SLEV)

St. Louis encephalitis virus (SLEV) was first discovered as the mosquito-borne agent responsible for over 1,000 cases of encephalitis during a 1933 summer outbreak in St. Louis (Missouri) and now is a reemerging human pathogen widely distributed in the American continent, causing several human encephalitis outbreaks over the last 80 years. Additional epidemics have indeed occurred from 1964 to 2006 in the Americas, ranging from the US to Argentina and Brazil. As a member of the *Flavivirus* genus, the SLEV E glycoprotein ectodomain is 68% identical to serocomplex-related JEV E but only 46% and 40% identical to those of DENV2 and TBEV, viruses from different serocomplexes [115].

#### 2.9.1. mAbs against SLEV

In 1983, Roehrig et al. isolated twenty-one hybridomas producing murine mAbs specific for the E glycoprotein of SLEV, strain MSI-7. Serologic reactivities were initially determined by cross-reactivity indirect immunofluorescence assays using 22 strains of SLEV and 8 other related *flaviviruses*. Four groups demonstrating type-, subcomplex-, supercomplex-, and group-specific reactivity patterns were identified. Analysis of hemagglutination-inhibition and virus neutralization subdivided the cross-reactivity groups into eight epitopes (E-1a, b, c, d, E-2, E-3, and E-4a, b), one of them following localized in DII (4b) [52, 53]. 

Moreover, among the previously described isolated anti-WNV mAbs by Gould et al., the chimeric scFv-Fc 79, effectively neutralized also SLEV, resulting in >80% of PRNT80 when used at 5 *μ*g/mL [42].

## 3. *Alphaviruses*

Major human arboviruses in the Togaviridae family, Chikungunya (CHIKV) and Venezuelan equine encephalomyelitis (VEEV) viruses, belong to the *Alphavirus* genus, which is composed of viruses with an icosahedral nucleocapsid surrounded by a lipid envelope and glycoprotein spikes. The structural proteins of *alphaviruses* arise through co- and post-translational processing of a polyprotein encoded by a single, positive-stranded RNA producing the capsid (C), PE2, 6 K, and E1. The PE2 glycoprotein is a precursor containing the E3 glycoprotein fused to the amino terminus of the E2 envelope glycoprotein. The PE2 glycoprotein is followed by 6 K, a small membrane-associated protein, and E1, the second polypeptide component of glycoprotein spikes.

Trimerized heterodimers of the E1 and E2 viral glycoproteins form the surface spikes and contain determinants of viral tropism and virulence. The E3 glycoprotein acts as a signal for transport of PE2 across the membranes of the rough endoplasmic reticulum and may promote the formation and intracellular transport of E1-PE2 heterodimers to the cell surface. During transport to the cell surface, PE2 undergoes a maturational cleavage event by a furin-like protease to produce E2 and E3 [116].

The E2 glycoprotein promotes specificity of virus binding to the host cell surface and is a target of the humoral immune response. The E1 glycoprotein mediates fusion of the virion envelope with the membranes of acidified endosomes, allowing release of the nucleocapsid into the cytoplasm and the onset of viral replication. *Alphavirus* E1 shares no appreciable sequence identity to *Flavivirus* E protein. Despite differences in their amino acid sequences and arrangements on the viral particle, the structures of E and E1 are remarkably similar. Indeed, the conservation of three domains has been well documented in crystal structures of *flavivirus* E ectodomains from TBEV, DENV, JEV, and WNV, as well as *Alphavirus* E1 from Semliki Forest virus (SFV) and CHIKV. Abs to the E1 glycoprotein do not typically neutralize virus infectivity *in vitro* but can protect against lethal challenge in animals. 

By analogy with the prM protein of dengue *Flavivirus*, furin cleavage of PE2 may begin after transit of an acidic late component of the Golgi body, where E3 is thought to suppress the acid pH-triggered activation of glycoprotein E1 fusion capability. Protective anti-E3 mouse mAbs have also been described [117]. 

### 3.1. Chikungunya Virus (CHIKV)

The chikungunya virus (CHIKV) belongs to the Semliki Forest clade and was firstly isolated in 1953 in Tanzania during an epidemic outbreak, following occurred also in Asia and Africa. In the last five decades, several CHIKV outbreaks have been described both in Africa and Asia separated by gaps lasting from two to twenty years. In 2005-2006, about 300,000 cases out of 785,000 inhabitants were reported in La Réunion island, with a fatal prognosis for 237 of the patients [117]. Neither Europe nor the Americas have had outbreaks of CHIKV so far, except for imported isolated cases. Three genotypes of CHIKV are described: Asian, East/Central/South African (ECSA), and West African, with an amino acid identity spanning from 95.2% to 99.8%. Recent epidemics in Africa and Indian subcontinent were caused by strains belonging to the ECSA genotype. Transmitted by *Aedes* mosquitoes, CHIKV is maintained in the human population by human-mosquito-human transmission [1]. The disease is characterized by dengue-like symptoms such as chills, high fever, headache, and persistent myalgia and a further incapacitating arthralgia (from which the name chikungunya) which affects 40% of infected subjects. However, prognosis is rarely fatal [116].

There is currently no commercial vaccine and antiviral treatment for CHIKV, although some candidate vaccines have been tested in humans. In this regard, in 2000 US Army performed a Phase II clinical trial testing a live-attenuated CHIKV vaccine (TSI-GSD-218) derived from a 1962 strain (15561) of an outbreak in Thailand. Out of 58, every patient developed neutralizing Abs, and 5 lamented mild to moderate joint pain [117]. A phase III trial of this candidate vaccine is ongoing. Furthermore, a new formulation using virus-like particles was able to induce neutralizing Abs in macaques against different CHIKV strains [117, 118]. Indeed, it has been described that infection seems to elicit long-lasting protective immunity and cross-protection among CHIKV and other *alphaviruses*.

CHIKV entry into host cell is demonstrated to be mediated by envelope glycoproteins E1 and E2, which allow virus fusion to cell membrane in low pH conditions and recognition of an unknown cellular receptor, respectively [116, 119–121]. Even though the genome replication relies on an error-prone RNA-dependent RNA-polymerasis, CHIKV strains S27 and 05.115 Reunion showed in E1 and E2 a low amino acid variation of 0.68% and 3.3%, respectively, and such a low variability may be needed for an effective replication in two phylogenetically distant hosts [116, 122]. Recently, the crystal structure of E1 and E2 heterodimer has been resolved both at low pH and natural conditions, shedding light on E1 and E2 ectodomains' structure. In particular, similarly to *flaviviruses* E protein, E1 ectodomain is made of the N-terminal DI, the DII containing the fusion loop, and the DIII at the C-terminal. On the other hand, E2 ectodomain comprises the N-terminal domain A, the domain B supposed to interact with the host cell's unknown receptor, and domain C at the C-terminal. E1 DIII and E2 domain C are located close to the viral membrane [123, 124].

#### 3.1.1. mAbs against CHIKV

Warter et al. described 5F10 and 8B10, two human mAbs which strongly neutralized several CHIKV isolates *in vitro*, without cross-reactivity against other *alphaviruses* but to Onyong-nyong virus [56]. Mixed preparation of 5F10 and 8F10 did not show neither synergistic nor addictive effect *in vitro*, and studies upon escape mutants demonstrated that 5F10 mAb binds at the tip of the E2 domain B, while 8B10 recognizes residues close to E1 fusion loop and amino acids within E2 domain A, which form a transitional epitope under low pH conditions. The authors speculated that 5F10 and 8B10 may inhibit CHIKV entry and fusion to the cell membrane, respectively. It is worth noting that the previously mentioned escape mutants displayed mutations associated with reduced viral fitness *in vitro* even after 13 neutralization/amplification rounds. In the same study, cell-to-cell transmission was firstly demonstrated as an escape mechanism, enhanced by the E2 mutation R82G [122].

In further *in vivo* studies, 5F10 and 8B10 significantly delayed CHIKV-caused death of AGR129 mice both in prophylactic and therapeutic tests. Interestingly, in therapeutic treatment, these mAbs showed a synergistic effect when administered in combination, with the total amount of mAbs injected being the same of the single-mAbs. Possibly, these treatment did not allow the mice to survive CHIKV challenge due to Ig clearing and 6–10 days half-life usually described for human mAbs [125].

Recently, Pal et al. cloned thirty-six murine mAbs able to neutralize the ECSA La Reunion 2006 OPY-1 strain of CHIKV (CHIKV-LR), the majority of which also neutralize infection of other strains corresponding to the Asian and West African genotypes. In particular, mAb CHK-152 showed the highest and broadest neutralizing activity, with FRNT values indicating an IC50 value of 1 to 3 ng/mL depending on the viral strain used. Among the thirty-six mAbs, four (CHK-102, CHK-152, and CHK-166, CHK-263) could prevent lethality of immunodeficient* Ifnar−/−* C57BL/6 mice when administered one day before exposition, with CHK-152 and CHK-263 showing the ability to protect mice at the lowest dose (10 *μ*g). Therapeutic studies were performed on these mAbs administering a single dose of 100 *μ*g 24 hours after infection. The highest activity was described for CHK-166, able to protect 63% of the mice. Testing the combined activity of the mAbs in therapeutic studies, administration of CHK-166 plus CHK-152 in a dose of 250 *μ*g each turned out to be the most effective, protecting 71% of the *Ifnar−/−* C57BL/6 mice 60 hours after infection [57].

### 3.2. Venezuelan Equine Encephalomyelitis Virus (VEEV)

Venezuelan equine encephalomyelitis virus (VEEV) is maintained in a natural transmission cycle between mosquitoes and small rodents. The first documented outbreaks occurred in the 1930s, and several epidemics have been reported so far in Latin-American countries such as Venezuela, Colombia, Peru, Ecuador, Bolivia, Honduras, Mexico, and Panama with hundreds of thousands of human cases being reported and a case-fatality rate up to 1% during the 1969 Ecuador outbreak. Clinical manifestations of VEE are indistinguishable from Dengue [126]. Six serogroups (I–VI) are currently recognized within the VEEV complex. VEEV caused human and equine outbreaks in the Americas for nearly a century. Equine epizootics have high mortality (38–83%) leading to a high viremia followed by a lethal encephalitis and often to human epidemics involving thousands of cases and hundreds of deaths. Indeed, VEEV is infectious for humans by the airborne route and has been responsible for a number of laboratory infections. In humans, the disease is usually self-limiting with a febrile illness with 1–4% of cases progressing to severe encephalitis. It is a hazard to laboratory workers, has been developed as a biological weapon, and is a potential bioterrorist agent [127]. There are no antiviral drugs and vaccines licensed for the treatment of VEEV infection in humans. Indeed, although experimental live-attenuated vaccines have been developed (e.g., TC-83) with good levels of protection in equine and mice, in humans they may fail to give protection in the majority of cases [128]. Considering the protective role exerted by humoral immune response in animal models, it could be concluded that treatment with specific IgG may have a beneficial antiviral effect in human airborne infections with pathogenic strains of VEEV.

#### 3.2.1. mAbs against VEEV

Several works support that mAbs may protect against airborne VEEV as well as after airborne exposure to VEEV. Ab reactivity to both surface glycoproteins (E1 and E2) is associated with neutralization of the virus *in vitro* and passive protection against virus challenge. A series of anti-E1 and anti-E2 mouse and humanized mAbs have been described recently. Among them, Phillpotts et al. examined two anti-E2 murine mAbs, named 1A4A-1 and 1A3A-9, both having potent protective activity against subcutaneous VEEV challenge in mice. Both mAbs had a similar half-life (5.8 and 10.0 days) in mouse serum after a single intraperitoneal dose, suggesting that mAbs, delivered by this route at or around the time of VEEV infection, persists at high levels in the blood as well as secretions in respiratory transudation throughout the clinical course of the disease. In particular both mAbs, administered 24 h prior to airborne challenges had a substantial protective effect (90–100%). Treatment of mice, 2 or 24 h after airborne infection, with a single intraperitoneal dose of 100 *μ*g of 1A3A-9 mAb, led to approximately 50% survival. There was no beneficial effect when mAb treatment was delayed to 72 h. Moreover, there was evidence of synergy *in vitro* in PRNT, between 1A3A-9 and 1A4A-1, as has been demonstrated for other viruses and mAb pairs. However, no synergy was found in mouse protection when mAbs were delivered intraperitoneally as a mixture in equal parts, to animals challenged 24 h previously with VEEV [129].

In a subsequent study Phillpotts et al. reported two other protective mAbs. The 3B2A-9 mAb protected against all the serogroup I strains while 1A3B-7 mAb protected well against challenge with all of the viruses tested. Both 3B2A-9 and 1A3B-7 protected against airborne exposure to the IA/B serogroup virus strain Trinidad donkey (TrD). However, there was no evidence of synergistic protection when these mAbs were combined in equal proportions. An intraperitoneal dose of 10 *μ*g was sufficient to protect 50% of the mice with either mAb [130]. According to other data, the mechanism of protection did not appear to depend upon neutralization [131]. Similar results were obtained by O'Brien et al., which described a non-neutralizing mAb (IgG2a), named CUF37-2a, able to protect 50% of mice from a subcutaneous VEEV challenge when a dose of 9.15 *μ*g of mAb was administered 24 h prior to challenge [132].

In a following study the 1A3B-7 mAb was humanized (and following reported as Hu1A3B-7) maintaining the same features of its murine counterpart. In particular, evaluation of *in vitro* studies indicated that Hu1A3B-7 retained both broad specificity and neutralizing activity. Furthermore, *in vivo* experiments showed that Hu1A3B-7 successfully protected mice against lethal subcutaneous and aerosol challenges with VEEV strain TrD. Moreover, the effectiveness of the humanization process was determined by assessing proliferation responses in human T-cells to peptides derived from the murine and humanized versions of the VH and VL domains. This analysis showed that the number of human T-cell epitopes within the humanized Ab had been substantially reduced, indicating that Hu1A3B-7 may have low immunogenicity *in vivo *[133].

Similarly, Hunt et al. described a humanized murine mAb derived from the 3B4C-4 mAb, recognizing the E2c epitope (amino acid residues 182–209) and following named Hy4-26C, showing neutralizing activity similar to the murine mAb. Moreover, it was protective (70–100% of survival) at 100 ng dose in mice animal model intraperitoneal inoculated and at 500 *μ*g for intranasal challenge (80%). Therapeutic studies in mice revealed that Hy4-26C was able to cure mice up to 24 h following infection at 10 *μ*g, on the contrary the mouse mAb when given 1 hour after virus challenge [63]. 

In another work, Hunt et al. isolated two neutralizing humanized Fabs, F5 and L1A7, employing a blocking strategy [134, 135]. The anti-E2 specific F5 IgG had a 70% PRNT endpoint of 10 ng/mL, equivalent to that described for the most effective neutralizing anti-VEEV E2 mAbs. In particular, the E1-specific hFab L1A7 had a PRNT endpoint of 3 *μ*g/mL, 300-fold lower than F5. 

Further studies revealed that F5 epitope is located in the 115–199 amino acid region. Moreover F5 IgG had potent ability to protect mice from infection by either route when administered 24 h before exposure; however, mice treated 24 h and 48 h after aerosol exposure developed central nervous system infections but exhibited no clinical signs of disease [135]. 

Hu et al. reported that passive immunization with the humanized chimeric mouse mAb hu1A4A1IgG1-2A in mice at 50 *μ*g 24 h before or after virulent VEEV challenge provided complete protection, indicating that hu1A4A1IgG1-2A has potent prophylactic and therapeutic effects against VEEV infection [136].

Anti-VEEV mAbs isolated from a non-human primate gene library has also been reported. In particular the humanized chimeric scFv-Fc ToR67-3B4 recognized viable as well as formalin and *β*-propiolactone inactivated virus particles. It detected specifically the viral E1 envelope protein of VEEV but did not react with reduced viral glycoprotein preparations suggesting that recognition depends upon conformational epitopes. The recombinant Ab was able to detect multiple VEEV subtypes and displayed only marginal cross-reactivity to other *Alphavirus* species except for Eastern equine encephalitis virus (EEEV). In addition, the scFv-Fc fusion described here might be of therapeutic use since it successfully inactivated VEEV in a murine disease model. In particular, when the recombinant Ab was administered 6 hours after challenge, 80% to 100% of mice survived lethal VEEV IA/B or IE infection. Forty to sixty percent of mice survived when scFv-Fc ToR67-3B4 was applied 6 hours after challenge with VEEV subtypes II and former IIIA [137].

## 4. Bunyaviridae

The Bunyaviridae family contains human arboviruses belonging to the *Orthobunyavirus*, *Phlebovirus*, and *Nairovirus* genera. Bunyaviridae includes enveloped viruses with a fragmented, single-stranded RNA genome of negative polarity. Their tripartite genome consists of a small (S), a medium (M), and a large (L) fragment and the envelope glycoproteins Gn and Gc that are cleaved out of a polyprotein synthesized by the M fragment. These glycoproteins have been proved to mediate the formation of the virus particle, to play a role in the interaction with cell surface receptors, to mediate the entry of the virus into cells, and to serve as targets for the majority of neutralizing Abs described so far. 

*Orthobunyavirus*, transmitted through mosquitoes or midges vectors, are divided in 18 serogroups, based on cross-titrations in haemagglutination inhibition assays and neutralization assays, and correlating with main vector preferences. However, the most clinically important viruses belong only to two serogroups, the California encephalitis and the Simbu serogroups.

Crimean-Congo hemorrhagic fever virus (CCHFV) is considered the only one of clinical relevance within the *Nairovirus* genus, which uses tick as main vector and is divided in seven serogroups, while Toscana virus and Rift Valley fever virus (RVFV), which are transmitted by sandflies and mosquitoes, respectively, belong to the *Phlebovirus* genus [138].

MAbs against the *Nairovirus* CCHFV and the *Phlebovirus* RVFV have been described.

### 4.1. Crimean-Congo Hemorrhagic Fever Virus (CCHFV)

Crimean-Congo Hemorrhagic Fever Virus (CCHFV) distribution covers the greatest geographic range of any tick-borne virus known, as viral isolation and/or disease has been reported from more than 30 countries in Southeastern Europe, Africa, Asia, and Middle East [139, 140]. Namely, from 1953 to 2010, about 6,000 human cases were reported in Southeastern Europe. CCHFV causes sporadic outbreaks with mortality rates ranging from 10 to 80% [141, 142]. A significant variation in the time of incubation has been described [139]. Prehemorrhagic symptoms include high fever, chills, headache, photophobia, and back and abdominal pains. Among other symptoms, neuropsychiatric changes have been reported in some patients. In severe cases, 3–6 days after the onset of the disease, hemorrhagic symptoms occur with petechiae, ecchymosis, bleeding in the form of melena, hematemesis, epistaxis [139].

In comparison with the nucleocapsid proteins, in CCHFV Gn and Gc show a higher degree of antigenic variability probably due to their exposition to the host immune system, hypothesis corroborated in the description by Hewson et al. in 2004 of four M segment phylogenic groups, namely, M1, M2, M3, and M4 [141, 142].

#### 4.1.1. mAbs against CCHFV

Blackburn et al. firstly described in 1987 murine anti-CCHFV mAbs able to recognize nucleocapsid proteins, but no neutralizing activity has been described thus far [143].

On the other hand, Bertolotti-Ciarlet et al. produced a panel of murine mAbs recognizing conformational epitopes within the Gn and Gc glycoproteins expressed by the CCHFV strain IbAr10200. The effectiveness of the mAbs was studied by performing PRNT80 and none of the anti-Gn mAbs showed neutralizing activity, although many of the anti-Gc mAbs neutralized IbAr10200 strain *in vitro* with clones 8A1, 5E3, 12A9, 6C2, and 9H3 showing activity at a >5120 dilution. MAbs 11E7 and 30F7 exhibited neutralizing activity at 2560 dilution. A suckling mice protection test was therefore performed for these mAbs. The previously mentioned anti-Gc Abs were capable to protect mice to an appreciable degree when applied 24 hours before and, in a weaker manner, 24 hours after virus challenge. Many of the anti-Gn mAbs were able to confer significant protection to IbAr10200 both 24 hours before and after virus administration. The relevant effectiveness of the anti-Gn mAbs 6B12, 10E11, 13G8, and 10G4 suggests that these mAbs may possess some neutralizing activity due to Ab-based effector mechanisms (e.g., ADCC) [58].

In further studies, the same group characterized the broadly cross-reactive neutralizing activity of the previously described murine mAb 11E7, able to bind conformational epitopes within the C-terminal region of the Gn glycoprotein of all CCHFV M groups [58, 141].

### 4.2. Rift Valley Fever Virus (RVFV)

Similarly to CCHFV, Rift Valley Fever Virus (RVFV) causes sporadic outbreaks. The largest epidemic occurred in Egypt in 1977, with an estimated 200,000 infections, 18,000 patients manifesting symptoms and 600 fatal prognoses [144]. Symptoms include hemorrhagic episodes, fever, encephalitis, and blindness [145]. 

The major target of the humoral immune response is the RVFV glycoproteins Gc (G1) and Gn (G2), ranging from nucleotides 480–2090 and 2091–3614, respectively [144]. 

#### 4.2.1. mAbs against RVFV

Within G2 glycoprotein are described three neutralizing antigenic sites, namely, epitope I: nucleotides 792–893 (amino acid residues 258–291); epitope II: nucleotides 1164–1196 (amino acid residues 382–392); and epitope IV: nucleotides 858–917 (amino acid residues 280–299) [59, 145]. 

Despite the high degree of variability observed in CCHFV glycoproteins, RVFV G2 presented an unexpected high conservation among 22 isolates [59]. In the same study, only the LUNYO and 900060 isolates were resistant to *in vitro* neutralization by murine mAb 4D4 (recognizing epitope II). All other isolates were neutralized with PRNT80 titers from 10,240 to >81,920. On the other hand, only SNS isolate showed reduced sensitivity to 4–39-CC (epitope IV). All other isolates were neutralized with PRNT80 titers from 20,480 to >81,920. Protection studies on mice would be of interest for the development of a passive immunization prophylaxis [59].

Protection studies from lethal South African RVFV AN1830 strain infection were successfully performed on a battery of anti-G1 and anti-G2 mAbs produced by Besselaar and Blackburn, with the strongly neutralizing anti-G1 mAb 3E5 and anti-G2 mAb 9C4 being the most effective [146]. The 9C4 antigenic area maps within G2 epitope I, and a broadly neutralizing activity may be investigated.

## 5. Conclusions

Arbovirus infections have acquired increasing interest given the augmented globalization and tourism movement all over the world that could be at the basis of epidemic events. Furthermore, the same vectors can sometimes transmit several arboviruses concomitantly, complicating the diagnosis as well as the therapy. For example, confusing mixed epidemics have occasionally been described, such as YFV plus CHIKV, DENV plus CHIKV, or more recently *Plasmodium falciparum* malaria plus DENV1 and CHIKV on Madagascar's east coast [1].

In the last decade, several efforts have been employed in the development of effective immunogens and therapeutics giving the current absence of specific antiviral drugs as well as effective vaccines for the most diffused arbovirus infections (i.e., DENV and WNV).

However, at the moment, mosquito control is the best available method for preventing arbovirus infections. Thus, to control the emerging public health problem of arbovirus infections, new antiviral therapeutic strategies that provide potent, and broadly cross-protective immunity (especially for *flavivirus* infections) are an urgent globally medical need.

As described in this review the majority of highly neutralizing mAbs are of murine origin and indeed their utility in the treatment of these infections is limited. In fact, their employment can elicit an immunogenic response (i.e., human anti-mouse Abs, HAMA), the therapeutic efficacy could be reduced by a relatively faster clearance in humans (compared to human Abs), potentially exacerbated by the HAMA response. Finally, murine Abs exhibit relatively weak effector functions (e.g., ADCC) compared to human Abs. Indeed, chimerization, humanization, or better, the isolation and characterization of fully human broadly neutralizing mAbs would be the best choice for their possible use in a future mAb-based therapy against arbovirus infections [147]. 

Finally, possible ADE of infection mechanisms should be evaluated before considering a mAb as a possible candidate therapeutic in a post-exposure setting as well as in the development of new Ab-eliciting immunogens.

## Figures and Tables

**Table 1:** Schematic summary of the best characterized mAbs against arboviral pathogens.

mAb	Origin	Reactivity	Target protein	Epitope	Cloning strategy	Format/isotype	Neutralizing activity	Reference
4.8A	Human	DENV1–4	E	DII	EBV transformation	IgG	IC50 (*μ*g/mL): DENV1: 1.5 DENV2: >40 DENV3: 2.4 DENV4: >40	[37]
D11C							IC50 (*μ*g/mL): DENV1: 1.5 DENV2: 1.0 DENV3: 10.2 DENV4: 1.6	
1.6D							IC50 (*μ*g/mL): DENV1: 1.5 DENV2: 0.2 DENV3: 0.5 DENV4: 2.7	

2H12	Murine	DENV1–4	E	DIII (AB loop: aa 314–317)	Hybridoma from BALB/c mouse immunized with DENV2 E/DIII	IgG2b	IC50 nM: DENV1: 0.56–54 DENV3: 29 DENV4: 145	[38]

C9	Murine/chimeric	DENV2	E	DIII	Phage display of a chimeric murine hybridoma library	VH1/V*κ*1	PRNT50 850 *μ*g/mL	[39]

4E11	Murine	DENV1–4	E	DIII (strand A: 308, 312 and strand G: 387, 389, 391)	Hybridoma	IgG2a/*κ*	IC50 (*μ*g/mL): DENV10.16 DENV2: 0.13 DENV-3: 8 DENV-4: 15	[40]

4G2	Murine	DENV1–4	E	DII (fusion loop)	Hybridoma from DENV2 E immunized mice	IgG	PRNT50 (*μ*g/mL): DENV2: 15	[39]

9F12	Murine	DENV1–4, WNV	E	DIII (aa 305, 307, 310, 330; A strand and BC loop)	Hybridoma from BALB/c mouse immunized with DENV-2 E/DIII	IgG1k	DENV1–4PRNT50: 2 · 10^−8^–2 · 10^−7^ M	[41]

2A10G6	Murine	DENV1–4, YFV, WNV, JEV, TBEV	E	DII (Fusion loop: aa 98–101)	Hybridoma from BALB/c mouse immunized with inactivated DENV2	IgG1	PRNT50 (*μ*g/mL): DENV1: 2 DENV2: 1.5 DENV3: 2.1 DENV4: 1.8 YNF: 3.6 WNV: 46	[36]

mAb11	Human	DENV1–4, WNV, SLEV, YFV, JEV MVEV	E	DII (fusion loop)	Phage display of human scFv	Fusion protein scFv-Fc	PRNT80 (*μ*g/mL): WNV: 1.25 DENV2: 6.25	[42, 43]

E16 (MGAWN1)	Murine	WNV	E	DIII (LR; aa 302–309)	Hybridoma from immunized mice with WNV E	IgG2b/humanized	PRNT50: 4–18 ng PRNT90: 53–297 ng	[44, 45]

1A1D-2	Murine	DENV1–3	E	DIII (A strand: aa 307, 310 and 312)	Hybridoma from immunized mice with different pH-treated virus	IgG2a	DENV2 PRNT50: 2.1 nM	[46, 47]

1F4	Human	DENV1	E (virion)	DI-DII	Electrofusion of infected memory B cells from DENV-immune subjects with EBV	IgG	DENV1 IC50 0.11 *μ*g/mL	[48]
2D22		DENV2		DIII			DENV2 IC50 0.08 *μ*g/mL	
5J7		DENV3		DI-DII			DENV3 IC50 0.10 *μ*g/mL	

E105	Murine	DENV1	E	DIII (BC loop: G328, T329 and D330; DE loop: K361E and E362K; FG loop: K385)	Hybridomas derived from C57BL/6 IFN-*αβ*R^−/−^ mice infected with DENV1	IgG	PRNT50 DENV-1: 0.5–59.2 ng/mL	[32]
E106				DIII (BC loop: G328, T329 and D330; DE loop: K361E and E362K; FG loop: K385; A strand: S305, K307, E309, K310, and E311)			PRNT50 DENV-1: 0.6–59.2 ng/mL	
E111				DIII (CC′ loop)			PRNT50: 3.8–25 *μ*g/mL	[49]

CR4374	Human	WNV	E	DIII	Phage display of scFv IgG library	VH2-05/VL1e	PRNT50: 0.18 *μ*g/mL PRNT90: 0.95 *μ*g/mL	[50]
CR4353						VH3-30/Vk3-A27	PRNT50: 0.026 *μ*g/mL PRNT90 36.4 *μ*g/mL	

1A5	Chimpanzee	DENV1–4, WNV, JEV, LGTV	E	DII (aa G106)	Phage display of Fab library from DENV1–4 infected chimpanzees	Humanized IgG1	PRNT50 (*μ*g/mL): DENV1: 0.48 DENV2: 0.95 DENV3: 3.2 DENV4: 4.3 WNV/DENV4: 3.8 JEV: 21 LGTV: 28	[51]

mAb11	Human	DENV1–4, WNV	E	DII (fusion loop, W101, G104, G106)	scFv library	scFv-Fc	PRNT80 (*μ*g/mL): DENV2: 6.25 WNV: 1.25	[42, 43]

3B4C-4	Murine	SLEV	E	1a	Hybridoma	IgG	SLEV PRNT: <1.7	[52, 53]
1B2C-5		SLEV		1b			SLEV PRNT: <1.7	
6B5A-2		SLEV		1c			SLEV PRNT: 4.8	
4A4C-4		SLEV		1d			SLEV PRNT: 2.9	
1B5D-1		SLEV, JEV		2			SLEV PRNT: <1.7	
2B5B-3		SLEV, JEV, MVEV, WNV, YFV		3			SLEV PRNT: 2.3	
2B6B-2		All *Flavivirus *		4a			SLEV PRNT: <1.7	
6B6C-1		All *Flavivirus *		4b (in DII)			SLEV PRNT: 2.3	

A3	Chimpanzee	JEV	E2	DI aa. K179	Phage display	Humanized	PRNT50 0.04–0.2 nM	[54]
B2				DII aa. I126	Phage display		PRNT50 0.02–2 nM	
E3				DIII aa. G132	Phage display		PRNT50 0.14–0.93 nM	

FabTJE12B02	Human	JEV	E	N/A	Phage display	Fab	FRNT50 50.2 *µ*g/mL	[55]

5F10	Human	CHIKV	E2	Domain B	EBV transformation	IgG1***λ***2	IC50 < 100 ng/mL	[56]
8B10	Human	CHIKV	E1-E2	E2 Domain A	EBV transformation	IgG1k	IC50 < 100 ng/mL	

CHK-152	Murine	CHIKV	E2	aa 59	Hybridoma	IgG2c	IC50 1–3 ng/mL	[57]

11E7	Murine	CCHFV	Gn	C-ter	Hybridoma	IgG	PRNT80 diluted 1/2560	[58]

4-39-CC	Murine	RVFV	G2	Domain IV	Hybridoma	IgG	PRNT80 diluted 1/20480–1/81920	[59]

2C9	Murine	YFV	E		Hybridoma	IgG2a	PRNT90: 1/10^4^ 17D 1/10^5.2^ Asibi	[60]

5A 7A R3(27)	Human	YFV	E	DI-DII	Phage display	ScFv	PRNT90: 0.1–0.3 *μ*g/mL	[61]

13D6	Murine	TBEV	E	DIII	Hybridoma	IgG/chimeric	PRNT50: 1.9 *μ*g/mL FRNT50: 4.5 *μ*g/mL IC50 1.9–16.7 *μ*g/mL	[62]

3B4C-4 (Hy4-26C)	Murine	VEEV	E2	aa 182–209	Hybridoma	IgG/humanized	PRNT70 (*μ*g/mL): 39.4–125	[63]
